# Kinetic modelling of myocardial necrosis biomarkers offers an easier, reliable and more acceptable assessment of infarct size

**DOI:** 10.1038/s41598-020-70501-4

**Published:** 2020-08-12

**Authors:** Stéphanie Chadet, David Ternant, François Roubille, Theodora Bejan-Angoulvant, Fabrice Prunier, Nathan Mewton, Gilles Paintaud, Michel Ovize, Anne Marie Dupuy, Denis Angoulvant, Fabrice Ivanes

**Affiliations:** 1 Loire Valley Cardiovascular Collaboration, Université de Tours, EA 4245 T2I & FHU SUPORT, Tours, France; 2 Laboratory of Pharmacology-Toxicology, CHRU de Tours, Tours, France; 3 Department of Cardiology, PhyMedExp, Université de Montpellier, INSERM U1046, CNRS UMR 9214, CHU de Montpellier, Montpellier, France; 4 Department of Clinical Pharmacology, CHRU de Tours, Tours, France; 5 Université d’Angers, EA 3860 CRT, Angers, France; 6 Université Claude Bernard Lyon 1, INSERM U1060 CarMeN, Lyon, France; 7 Department of Cardiology & FACT, CHRU de Tours, Tours, France

**Keywords:** Cardiovascular diseases, Prognostic markers, Translational research

## Abstract

Infarct size is a major prognostic factor in ST-segment elevation myocardial infarction (STEMI). It is often assessed using repeated blood sampling and the estimation of biomarker area under the concentration versus time curve (AUC) in translational research. We aimed at developing limited sampling strategies (LSS) to accurately estimate biomarker AUC using only a limited number of blood samples in STEMI patients. This retrospective study was carried out on pooled data from five clinical trials of STEMI patients (TIMI blood flow 0/1) studies where repeated blood samples were collected within 72 h after admission to assess creatine kinase (CK), cardiac troponin I (cTnI) and muscle-brain CK (CK-MB). Biomarker kinetics was assessed using previously described biomarker kinetic models. A number of LSS models including combinations of 1 to 3 samples were developed to identify sampling times leading to the best estimation of AUC. Patients were randomly assigned to either learning (2/3) or validation (1/3) subsets. Descriptive and predictive performances of LSS models were compared using learning and validation subsets, respectively. An external validation cohort was used to validate the model and its applicability to different cTnI assays, including high-sensitive (hs) cTnI. 132 patients had full CK and cTnI dataset, 49 patients had CK-MB. For each biomarker, 180 LSS models were tested. Best LSS models were obtained for the following sampling times: T4–16 for CK, T8–T20 for cTnI and T8–T16 for CK-MB for 2-sample LSS; and T4–T16–T24 for CK, T4–T12–T20 for cTnI and T8–T16–T20 for CK-MB for 3-sample LSS. External validation was achieved on 103 anterior STEMI patients (TIMI flow 0/1), and the cTnI model applicability to recommended hs cTnI confirmed. Biomarker kinetics can be assessed with a limited number of samples using kinetic modelling. This opens the way for substantial simplification of future cardioprotection studies, more acceptable for the patients.

## Introduction

Infarct size (IS) is a key predictor of subsequent major cardiovascular events in the context of myocardial ischemia–reperfusion injury^1, 2^. Its utilization has been for years a surrogate major objective of phase II cardioprotection studies^3–5^. The gold standard of IS assessment is the quantification of late gadolinium enhancement in cardiac magnetic resonance imaging (MRI). Since access to MRI is often limited in routine practice and biplane left ventricular angiography significantly increases the volume of iodinated contrast media (and the risk severe induced ventricular arrhythmias), serial blood measurements of necrosis biomarkers are classically used as surrogate endpoints in trials in the field of cardioprotection^6^, notably serum creatine kinase (CK), creatine kinase muscle-brain specific of cardiomyocytes (CK-MB) and troponins^7, 8^. Up to date, because of their availability and their known correlation with IS^9–11^, the total amount of necrosis biomarker is estimated using the peak and/or the area under the concentration versus time curve (AUC) of CK, CK-MB and troponins, determined using trapezoid method^12^.

However, in order to obtain accurate evaluation of IS by AUC, investigators are prompted to perform a large number of repeated blood samples (12 to 16 within 72 h instead of generally two measurements on the day of admission and one daily afterward in routine clinical practice)^13, 14^. Repeated blood sampling in patients after ST-segment elevation myocardial infarction (STEMI) may be perceived as a burden for patients, generating discomfort and reluctance to clinical research, and both paramedics and investigators, leading to increased cost and risk of missing data.

In a previous study, we developed and validated mathematical models to describe the serum kinetics of CK, CK-MB and troponins, using individual data from five clinical trials that assessed the efficacy of conditioning therapies in STEMI^13–19^. In this context, decreasing the number of samples without loss in estimation accuracy is a major challenge and is, of course, not possible with the classical trapezoidal rule AUC approach. The need for limited sampling strategies (LSS) to describe individual kinetic profiles led to development of Bayesian estimators (BE) of kinetic parameters^20^. This approach has been widely used in therapeutic drug monitoring, notably of immunosuppressant drugs such as mycophenolate^21–23^ and allowed significant reduction of blood samples in this field (3 samples). We hypothesized that such a Bayesian approach may be used to estimate myocardial biomarker AUC in STEMI, and thus IS, without any significant loss of accuracy. Our mathematical models^15^ should allow for the first time the estimation of biomarker AUC using a small number of blood samples (typically 2 or 3 samples per patient) and provide to physicians easy and reliable access to infarct size data.

The aim of the present study was therefore (i) to develop LSS allowing the estimation of necrosis biomarker (CK, CK-MB, troponins) AUC based on recently published biomarker kinetics models, (ii) to compare these LSS to full kinetic sampling profiles, and (iii) to provide the best sampling times for these estimations using our kinetic models.

## Results

Among the 246 patients included in the 5 clinical trials, 181 patients had a full biomarker measurement profile and their data were used for this work (Table 1). The full data sets, i.e. with CK, cardiac troponin I (cTnI) and CK-MB, included 132, 132 and 49 patients, respectively. In learning and validation subsets, 84 and 48 patients were assessed for CK and cTnI, respectively, whereas 32 and 17 patients were respectively asessed in learning and validation subsets for CK-MB.

**Table 1:** Summary of assessed patients’ characteristics.

Study parameters	CK and cTnI	CK-MB	External validation cohort
Patients evaluable	132	49	103
Gender (male/female)	101/31	40/9	85/18
Study arms	Control—52	Control—16	–
	Conditioning—61	RIPer—17	–
		RIPer + IPOST—16	
Age (years)	56 (49–68)	59 (49–72)	62 (53–73)
Active smokers (%)	57.8	36.4	38.3
Arterial hypertension (%)	43.9	36.4	52.3
Body Mass Index (kg/m^2^)	25.95 (23.3–29.1)	25.95 (23.03–28.56)	25.35 (23.94–27.43)
Dyslipidaemia (%)	47.4	40	51.4
Diabetes mellitus (%)	16.8	5.5	14
eGFR (MDRD formula, mL/min/1.73 m^2^)	85.5 (70.25–98.75)	82.4 (62.65–98.5)	86.2 (71.6–103.7)
Area at risk (% of ACS)	34.7 (27.0–46.5)	37.4 (30.5–45.7)	–
LVEF (%)	50.7 (43–61)	48.2 (44.1–53.2)	44 (35–54)
AUC CK (IU h/L)	74,212 (32–1,09,236)	–	
CK peak (IU/L)	2,745 (1,671–4,418)	–	
AUC cTnI (mg h/L)	2,607 (1,452–4,267)	–	
cTnI peak (mg/L)	97 (46–161)	–	
AUC CK-MB (IU h/L)	–	5,403 (3,631–7,742)	
CK-MB peak (IU/L)	–	306 (196–451)	

*ACS* abnormally contracting segments, *AUC* area under the concentration versus time curve, *CK* creatine kinase, *CK-MB* creatine kinase muscle-brain specific of cardiomyocytes, *cTnI* cardiac troponin I, *eGFR* glomerular filtration rate, *IPOST* ischemic post-conditioning, *LVEF* left ventricular ejection fraction, *MDRD* modified diet and renal disease, *RIPer* remote ischemic per-conditioning.

### Biomarker LSS models accurately described biomarker AUC

**Figure 1:**
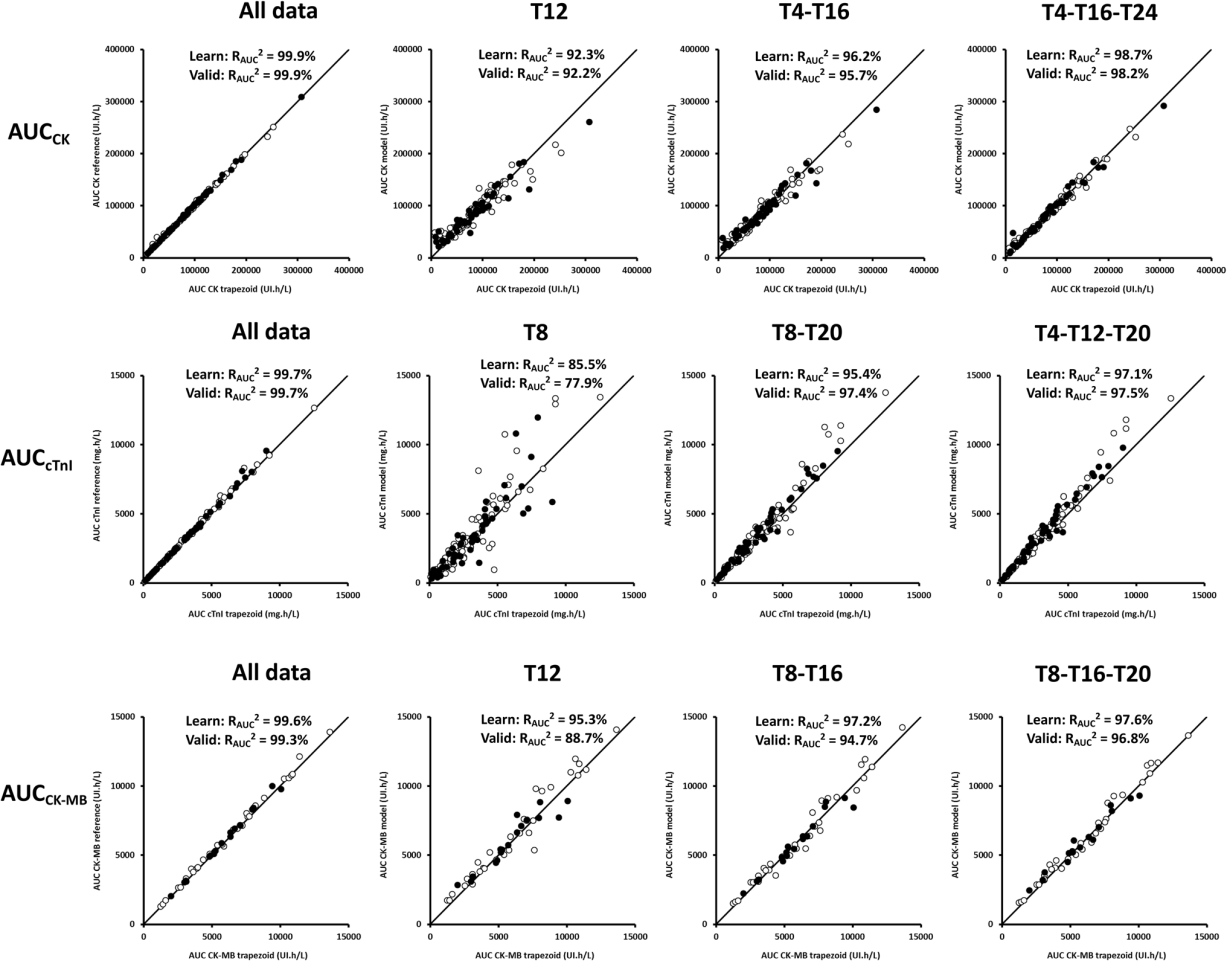
Observed *versus* model-predicted creatine kinase (CK, up), cardiac troponin I (cTnI, middle) and creatine kinase muscle-brain (CK-MB, bottom) for limited sampling strategies. From left to right, all data points, then best 1-sample, 2-sample and 3-sample limited sampling strategies. Open and dark circles are observed/predicted biomarker level couples for learning an validation sets, respectively, the line is the first bisector line.

Biomarker kinetic models in the learning set satisfactorily described learning subset data of CK, cTnI and CK-MB. In addition, model performances in learning and validation sets were totally comparable (Fig. 1, Supplemental Data).

Overall, we ran 15, 45 and 120 LSS models with 1, 2 and 3 sampling times for each biomarker, respectively (Table 2). Details on LSS model development are presented in Supplemental Data.

**Table 2:** Summary of 1-, 2- and 3-sample limited sampling strategies.

Number samples	1 sampling time			2 sampling times			3 sampling times		
Biomarker	CK	cTnI	CK-MB	CK	cTnI	CK-MB	CK	cTnI	CK-MB
Number of LSS model tested	15	15	15	45	45	45	120	120	120
Number of LSS models with R^2^ > 90%	1	0	2	12	3	24	67	29	92
Sample times (hours)	T12	T8	T12	T4, T16	T8, T20	T8, T16	T4, T16, T24	T4, T12, T20	T8, T16, T20
R^2^ learning set (%)	92.3	85.5	95.3	96.2	95.4	97.2	98.7	97.1	97.6
R^2^ validation set (%)	92.2	77.9	88.7	95.7	97.4	94.7	98.2	97.5	96.8

*CK* creatine kinase, *CK-MB* creatine kinase muscle-brain specific of cardiomyocytes, *cTnI* cardiac troponin I, *LSS* limited sampling strategy, *R*^*2*^ coefficient of determination, *Tx* sampling at time x.

Among LSS models with R^2^ > 90%, relative bias < 10% and last sampling time ≤ 24 h, best LSS were: T12 for CK and CK-MB, and T8 for cTnI for one sample LSS; T4–16 for CK, T8-T20 for cTnI and T8–T16 for CK-MB for two sample LSS; and T4–T16–T24 for CK, T4–T12–T20 for cTnI and T8–T16–T20 for CK-MB for three sample LSS (Table 2, Fig. 1).

Regarding the association between models and area at risk of ischemic myocardium (AAR), the association of AAR was tested as a covariate on kinetic parameters. A significant association of the AAR with amount of biomarker release (B0) was found for CK and CK-MB, increased AAR resulted in increased B0 (Supplemental Data). LSS models were built on the basis of these associations.

### Biomarker LSS models accurately predicted biomarker AUC

**Figure 2:**
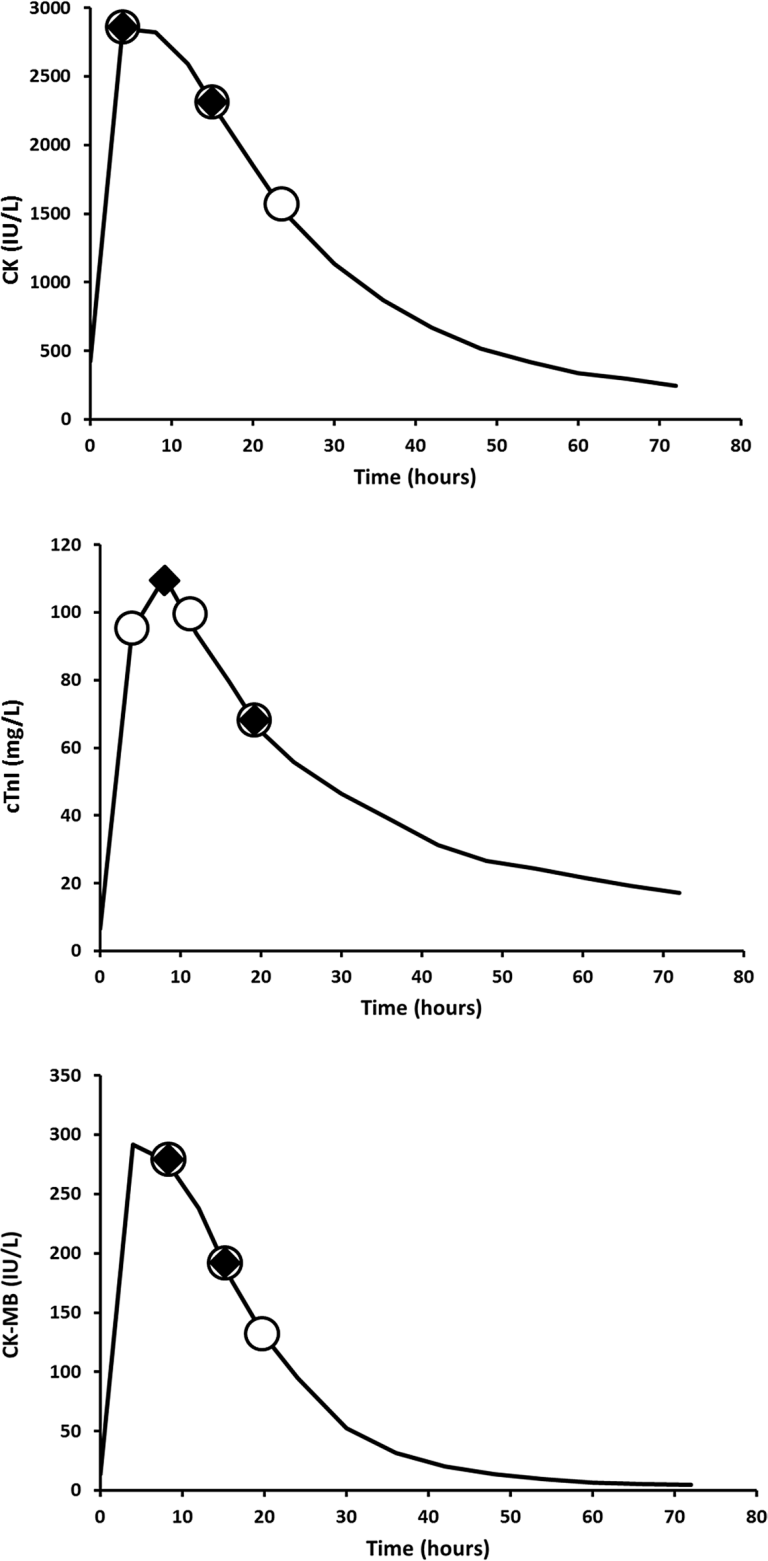
Sampling times for best 2-sample (full diamonds) and 3-sample (open circles) limited sampling strategies (LSS). Lines are median creatine kinase (CK), cardiac troponin I (cTnI) and muscle-brain creatine kinase (CK-MB) kinetic profiles vs. time. Best LSS were: T4–16 for CK, T8–T20 for cTnI and T8–T16 for CK-MB for 2-sample LSS; and T4–T16–T24 for CK, T4–T12–T20 for cTnI and T8–T16–T20 for CK-MB for 3-sample LSS.

The prediction performances of LSS models of the validation step were similar to those found during the development step (Table 2). The best LSS models determined previously were confirmed to accurately predict AUC of biomarkers (Table 2). For the three biomarkers, 3-sample LSS strategies led to better descriptive and predictive performance than 1 and 2-sample strategies. Therefore, best 3-sample strategies were T4–T16–T24, T4–T12–T20 and T8–T16–T20 for CK, cTnI and CK-MB, respectively (Fig. 2).

If we consider the best strategy gathering the three biomarkers, then the best times would be T4–T12–T20 with respective R^2^ of 95.6%, 92.8% and 94.0%. Of note, this is not the best strategy for either CK or CK-MB.

### External validation

**Figure 3:**
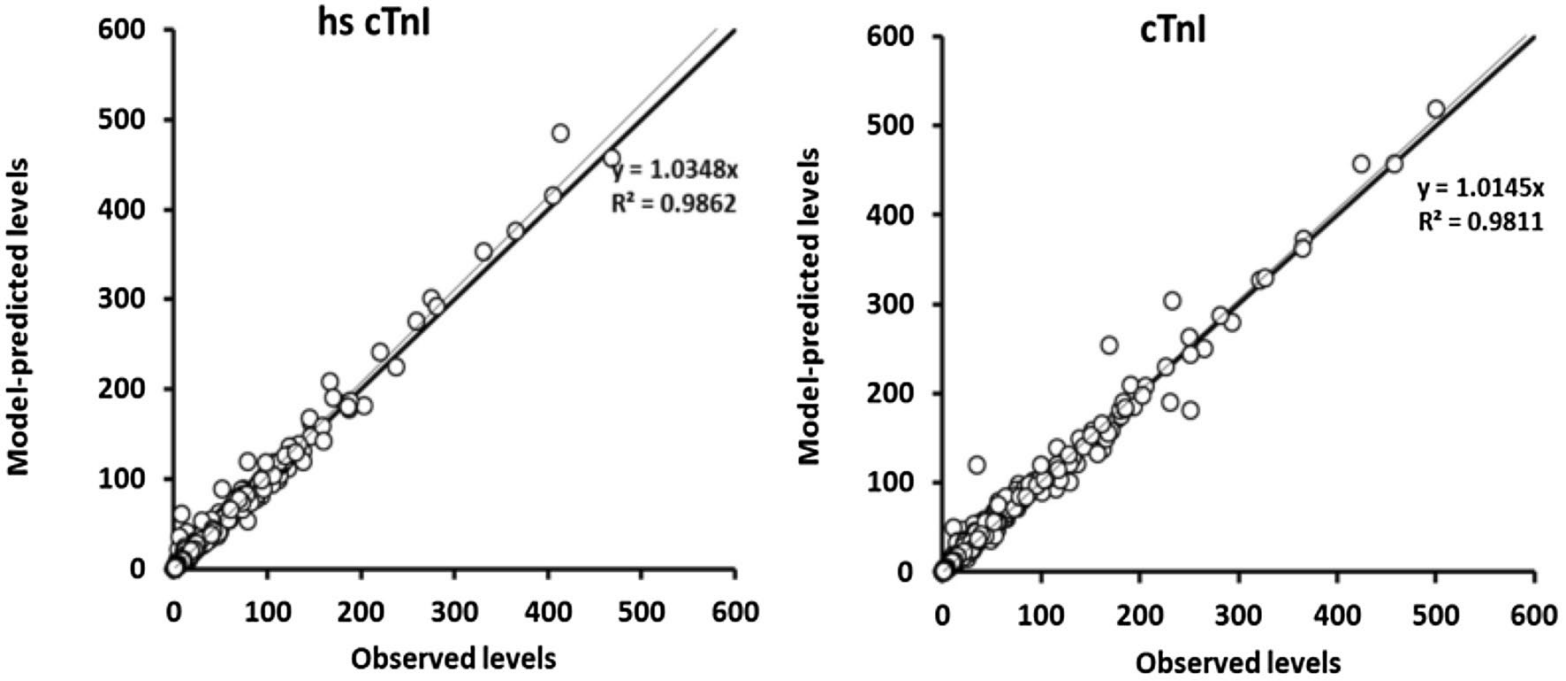
Observed vs. model-predicted biomarker levels (based on 484 observations of each biomarker). Left, hs cTnI assay from Abbott; right, conventional non-hs cTnI assay from Siemens.

484 cTnI samples taken in the first 24 h following admission for STEMI with TIMI 0/1 initial blood flow, from 103 patients allowed us external validation of the cTnI model. All these patients had both high-sensitive (hs) and non-hs cTnI samples data available for different time points. Detailed patients characteristics for this validation cohort are provided in Table 1 and can be retrieved in the publication from Laugaudin et al.^24^. Application of the cTnI model to both non-hs and hs cTnI samples showed excellent predictive performance of cTnI model, as depicted in Fig. 3.

The results regarding R^2^ and relative bias were:

$${\text{cTnI in the reference cohort}}\quad {\text{bias }} = - \;0.{2}\% \quad {\text{R2 }} = \, 0.{97}0$$

$${\text{cTnI in the external cohort}}\quad {\text{bias }} = { 1}.{5}\% \quad {\text{R2 }} = \, 0.{981}$$

$${\text{hs cTnI in the external cohort}}\quad {\text{bias }} = { 3}.{5}\% \quad {\text{R2 }} = \, 0.{986}$$

This further validates the cTnI model with regards to the different assays available, including hs cTnI assays.

## Discussion

This study is the first to propose a simple and powerful approach of necrosis biomarker data assessment based on a limited number of blood samples taken during a short delay (within 24 h) in ST-segment elevation myocardial infarction patients TIMI flow 0/1 revascularized by PCI. Based on our previous mathematical description of biomarker kinetics, we demonstrated that estimation of AUC of CK, cTnI and CK-MB biomarkers using 3-sample LSS strategies led to excellent biomarker AUC description and prediction. In addition, we provide external validation with regards to our cTnI model and its applicability to modern hs cTnI assays.

Based on our results, the overall optimal pattern would be to take the three samples between T4 and T24 with a delay of 4–6 h between samples. Sampling times should be respected since the accuracy of LSS models relies on the high information level carried by samples, as proposed above. If best description and prediction performances are given by 3-sample LSS, very good performances are still obtained using 2-sample LSS. For 2-sample LSS, sampling times should be T4–T16 for CK, T8–T20 for cTnI and T8–T16 for CK-MB (Fig. 2). Of note, for 3-sample LSS, the high number of LSS strategies leading to R^2^ > 90% suggests a certain flexibility in sampling times that should help reconnecting the requirements of clinical research with the reality of day-to-day patients’ care and still ensure good quality data collection. Besides, CK-MB appears to be more flexible than CK or cTnI because most of 3-sample LSS (more than 75%) led to very good predictive performance (R^2^ > 90%) compared to CK (approximately 50%) or cTnI (approximatively 25%). Paradoxically this biomarker is becoming less and less used and may not be available in every centre.

Our data demonstrated the excellent predictive performance of our models in the validation subset. Although we observed a non-neglectable decrease in predicting performance of CK-MB in the validation subset, we believe that this may be related to the small number of patients (17) with CK-MB measurements in that subset. In order to optimize the external validation of our models, it needs to be tested on databases totally independent from the present study. Importantly, both kinetic models and LSS should be performed without any change regarding model structure, parameter values, or sampling times.

Another research group published detailed analysis of necrosis biomarker kinetics in order to investigate differences between troponins I and T^24^. Yet, their analysis does not allow to draw a general model that can be applied outside their data set. Nor can it be used to reduce the number of samples. In addition, a model describing troponin kinetics was developed in order to quantify the toxicity of the association of trastuzumab and anthracyclins. However, since troponin input was linked to anthracyclin infusion, this model is not suitable for accurate AUC estimation in the context of STEMI^25^.

The added value of the Bayesian approach performed in this study is to provide a significant amount of information using limited number of samples in each patient. However, one must acknowledge that there is still a slight loss of information, estimated to be below 5% for best sampling strategies, that may be attenuated by the overall general performance of the model, which is more accurate in estimating the total amount of necrosis biomarker released than the trapezoid-calculated AUC itself^15^. These LSS models, applied to future cardioprotection studies in STEMI aiming at reducing infarct size, may greatly improve the research process, with a significant gain in human and financial resources for the investigators, and in comfort for the patients participating in such studies. The next validation step is to determine their correlation with the gold standard technique for infarct size assessment, i.e. late gadolinium enhancement in cardiac MRI, and confirm that this method is a suitable alternative to MRI for infarct size determination. Yet, this mathematical model will never be able to outperform MRI in all its post-infarction indication as it cannot give any indication regarding the presence of a left ventricular thrombus.

This work has however several limitations. Despite the fact that we pooled data from five clinical trials, the groups were of reduced size, in particular the CK-MB group. Another limitation is that our models are not defined for cardiac troponin T, as this biomarker was not available in our study population. Yet this biomarker, together with cTnI, is the main biomarker used for the definition of myocardial infarction and may be the only troponin assay available in many centers^8^. The adaptation of the model to troponin T measurements and kinetics is needed, and will be done on new patients’ databases. Our cTnI model was derived from non-hs cTnI from two different assays, yet with very similar characteristics. We provided here external validation of the model, both on another non-hs cTnI assay, and more interestingly on a newer hs-cTnI assay. With regard to these data, we are convinced of the ability of our model to describe cTnI kinetic data obtained from various (hs) assays, since Bayesian estimation of kinetic parameters is based on mixed-model effect modelling, which allows to take into account variability due to non-controlled or unknown factors, including differences in measurement techniques^20, 26–29^. Our kinetic models were developed on TIMI 0/1 STEMI patients and whether the models may be applicable to all-comers STEMI, including TIMI 2/3, requires further investigation. Similarly, our models were not tested on patients whom primary reperfusion treatment was intravenous fibrinolysis. More data are needed before we can validate our models in this specific population, as well as in patients with ongoing cardiogenic shock. Late presenter STEMI patients admitted after the primary PCI window may still benefit from this model-based infarct size assessment if the first blood sample is taken in the 72 h’ time limit. Yet, the model was not derived from these patients and might further loose in accuracy. This may also be the case in patients with failed angioplasty or severe renal insufficiency. Last, our models are not applicable to non-STEMI patients. Yet, these are completely different patients from STEMI patients as pointed out by most population studies, including the latest French registry FAST-MI^30^. In these patients, the prognosis is far more related to comorbidities and extent of coronary artery anomalies than to proper infarct size. Thus, infarct size is a less relevant parameter in this population.

## Conclusion

We demonstrated that biomarker kinetics can be assessed using a limited number of samples (2 or 3 samples, which means until 7 times less than in current protocols) thanks to Bayesian kinetic modelling. This opens the way for substantial simplification of future cardioprotection studies in TIMI 0/1 STEMI with more acceptable protocols for patients, research teams and investigators. This approach will increase power and put an end to the classical trapezoidal rule AUC. Bayesian analysis is likely to be a useful add-on to future studies in the field of cardioprotection. In addition, it offers a simple way and inexpensive to estimate infarct size for patients presenting with STEMI in routine clinical care.

## Methods

### Patients population and data collection

We used data from five clinical trials in STEMI patients (Supplemental Data) that evaluated the effect of a conditioning strategy (ischemic post-conditioning, pharmacological post-conditioning with cyclosporine A, remote ischemic per-conditioning) versus control for CK, cTnI^13, 16–19^ and CK-MB^14^. These trials were approved by local ethical committees and all patients provided written informed consent. All patients in these studies were admitted within 12 h of the onset of chest pain and had a TIMI 0/1 coronary blood flow at first culprit coronary artery injection. Primary endpoint was infarct size estimated by AUC of CK, CK-MB and non-hs cTnI, based on serial serum measurements. The AAR was estimated using a biplane left ventricular angiography in order to measure the circumferential extent of abnormally contracting segments (ACS)^31–34^. Blood samples were collected prior to primary percutaneous coronary intervention, every 4 h in the first 24 h following intervention, and every 6 h over the following 48 h. Therefore, 15 samples (T0, T4, T8,…, T72) were available for each patient and each biomarker. These samples had been used to compute AUC toward 72 h for every patient individually, for CK (AUC_CK_), cTnI (AUC_cTnI_) and CK-MB (AUC_CK-MB_). For both CK and non-hs cTnI, concentrations were measured using two distinct assays from Beckman Coulter (Villepinte, France) and then from Abbott (Rungis, France). For the first 54 patients, non-hs cTnI concentrations were measured using Accu kit on Access 2 system (Beckman Coulter) and CK concentrations were measured using CK reactive kit on Synchron LX system (Beckman Coulter). For the 78 remaining patients, non-hs cTnI concentrations were measured using STAT ARCHITECT kit on Architect I2000 system (Abbott) and CK concentrations were measured using Abbott 7D63 CK kit on Architect C8000 and C16000 instruments (Abbott). The kits had close characteristics: similar limits of detection (0.01 ng/mL), 99th percentile concentration (0.040 ng/mL and 0.028 ng/mL for Beckman Coulter and Abbott, respectively), coefficient of variation of 99th percentile (14%) and no significant difference in either concentration measurements, or in kinetic parameters were found between these assays (see Supplementary Material for detailed characteristics).

This study was conducted in accordance with the Declaration of Helsinki, approved by the institutional review board of the Pole Coeur Thorax Vaisseaux from the Tours University Hospital (Tours, France), and was registered as a clinical audit^15^. All data were fully anonymized and the present study was conducted retrospectively. Patients were not involved in its conduct, and there was no impact on their care.

### Data splitting

Measurements of cTnI and CK were pooled for kinetic modeling assessment. CK-MB measurements from the remote ischemic per-conditioning study were assessed separately. Three full data sets, i.e. with CK, cTnI and CK-MB variables, were built. With the aim of validating LSS models to estimate both biomarker input and AUC, data sets were randomly assigned (computerized allocation) into learning (2/3 patients) or validation (1/3 patients) subsets (Fig. 1). Learning and validation subsets were used for internal and external validation of LSS estimators, respectively. To ensure homogeneity between both subsets, the randomization of the study population was stratified on study (to avoid time bias) and treatment (conditioning or control).

We also seeked for external validation and therefore ran our models using individual data of another french cohort of 103 patients with anterior STEMI and a TIMI 0/1 blood flow. These patients were refered for primary percutaneous coronary intervention within 12 h of the onset of chest pain and had multiple biomarkers assessments from admission and every 12 h up to 132 h. cTnI was measured using different assays: a non-hs cTnI assay and a hs cTnI assay with the following characteristics.

Non-hs cTnI were measured using the Advia Centaur Ultra cTnI assay on an Advia Centaur Immunoassay analyser from Siemens, with a 10% coefficient of variation limit of 30 ng/mL and a 99th percentile of 40 ng/mL (confidence interval 20–60). Hs cTnI were measured using the Architect STAT High Sensitive Troponin-I Reagent on an Architect i1000SR from Abbott Diagnostics. The 10% coefficient of variation limit was 3.9 ng/mL and the 99th percentile values were 14 ng/mL for men and 11 ng/mL for women (see Supplementary Material for detailed characteristics).

We wanted to ensure that: (i) our cTnI model could be applied to STEMI patients others than those from our cohort, and (ii) that this model was extrapolable to others cTnI assays, including the newer and now recommended hs cTnI.

### Cardiac biomarker kinetics and LSS modeling

The kinetic models development is described in details elsewhere^15^. Briefly, these models described biomarker release, distribution and elimination steps and allowed computing AUC for CK, cTnI and CK-MB. Kinetic parameters were estimated using nonlinear mixed-effect modelling approach^20^.

Based on our kinetic models, the interindividual distribution of parameters and individual values of AUC were estimated in the learning data subset using all samples (T0 to T72). Assessment of the predictive performance of kinetic models was made by comparing computed biomarker AUC and AUC calculated by the reference method, i.e. trapezoid rule. This comparison was made numerically by comparison of model-estimated AUC with AUC calculated by trapezoid rule using coefficient of determination (R^2^, %) and relative bias (%), which is the systematic error of estimation relative to observation. A model was considered to provide accurate results if R^2^ > 90% and relative bias < 10%. Among LSS leading to similar performance, strategy with the lowest time of last sample was chosen, with all sampling times ≤ 24 h.

### Statistical analyses

Coefficients of determination and bias were used to assess the predictive performance of kinetic and LSS models. The analysis was made using R 3.2.3 (R Core Team, Vienna, Austria)^35^.

## Supplementary information

Supplementary Information

## Data Availability

The datasets used and/or analysed during the current study are available from the corresponding author on reasonable request.

## Abbreviations

AAR: Area at risk of ischemic myocardium
ACS: Abnormally contracting segments
AUC: Area under the concentration versus time curve
BE: Bayesian estimators
CK: Creatine kinase
CK-MB: Creatine kinase muscle-brain
cTnI: Cardiac troponin I
IS: Infarct size
LSS: Limited sampling strategy
MRI: Magnetic resonance imaging
STEMI: ST-segment elevation myocardial infarction
TIMI flow: Blood flow according to the thrombolysis in myocardial infarction classification
