# Supplementary material for: Kinetic modelling of myocardial necrosis biomarkers offers an easier, reliable and more acceptable assessment of infarct size
Source: Sci Rep. 2020 Aug 12;10:13597. doi: 10.1038/s41598-020-70501-4 (PMC7423884; doi:10.1038/s41598-020-70501-4)
Supplement: Supplementary file 1 — Supplementary Information [file 41598_2020_70501_MOESM1_ESM.pdf]

ADDITIONAL FILE for *Scientific Reports*

Kinetic modelling of myocardial necrosis biomarkers offers an easier, reliable and more acceptable assessment of infarct size

Stéphanie Chadet[1], PhD, David Ternant[2,3], PharmD, PhD, François Roubille[4], MD, PhD, Theodora Bejan-Angoulvant[2,5], MD, PhD, Fabrice Prunier[6], MD, PhD, Nathan Mewton[7], MD, PhD, Gilles Paintaud[2,3], MD, PhD, Michel Ovize[7], MD, PhD, Anne Marie Dupuy[8], MD, PhD, Denis Angoulvant[1,9], MD, PhD, Fabrice Ivanes[1,9]*, MD, PhD,

[1] Université de Tours, EA 4245 T2I & FHU SUPPORT, Loire Valley Cardiovascular Collaboration, Tours, France
[2] Université de Tours, CNRS, UMR 7292 GICC, Tours, France
[3] CHRU de Tours, Laboratory of Pharmacology-Toxicology, Tours, France
[4] PhyMedExp, Université de Montpellier, INSERM, CNRS, Cardiology Department, CHU de Montpellier, France.
[5] CHRU de Tours, Department of Clinical Pharmacology, Tours, France
[6] Université d'Angers, EA 3860 CRT, Angers, France
[7] Université Claude Bernard Lyon 1, INSERM U1060 CarMeN, Lyon, France
[8] University Hospital of Montpellier, Department of Biochemistry and Hormonology, Centre Ressources Biologiques de Montpellier, Montpellier, France
[9] CHRU de Tours, Department of Cardiology & FACT, Tours, France

SUPPLEMENTARY FIGURES/TABLES

Supplementary table 1. Kinetic parameter estimates

Biomarker		CK (IU/L)	cTnI (mg/L)	CK-MB (IU/L)
Parameter	Unit	Estimate (RSE %)	Estimate (RSE %)	Estimate (RSE %)
**B_{TOT}**	**Biomarker**	**2120 (23)**	**196 (29)**	**167 (18)**
Arm on B_{TOT}	–	-0.43 (34)	-0.56 (38)	-0.75 (29)
AAR on B_{TOT}	–	0.020 (30)	0.019 (39)	0.037 (24)
B_1	biomarker	173 (12)	0.11 (41)	43.1 (36)
B_2	biomarker	–	–	–
Fr	–	0.15 (31)	0.53 (14)	–
n_1	nb of transit compart.	2.5 (7)	2.0 (10)	1.0 (19)
k_{tr1}	h^{-1}	0.68 (11)	0.29 (6)	0.34 (12)
n_2	nb of transit compart.	50.7 (8)	4.2 (6)	–
k_{tr2}	h^{-1}	14.4 (7)	0.88 (1)	–
k_{prod}	Biomarker / h	6.9 (11)	–	0.32 (8)
k_e	h^{-1}	0.062 (3)	0.097 (6)	0.096 (2)
k_{12}	h^{-1}	–	0.11 (8)	–
k_{21}	h^{-1}	–	0.044 (12)	–
ω_{Btot} (-)	–	0.65 (8)	0.80 (8)	0.51 (14)
ω_{B1} (-)	–	1.1 (8)	3.7 (18)	1.1 (29)
ω_{B2} (-)	–	–	–	–
ω_{Fr} (-)	–	0.79 (20)	0.59 (13)	–
ω_{n1} (-)	–	0.49 (11)	0.46 (11)	0.64 (23)
ω_{Ktr1} (-)	–	0.78 (10)	0.43 (14)	0.18 (22)
ω_{n2} (-)	–	0 (fixed)	0.25 (27)	–
ω_{Ktr2} (-)	–	0 (fixed)	–	–
ω_{Kprod} (-)	–	0.83 (10)	–	0.37 (15)
ω_{Ke} (-)	–	0.23 (10)	0.23 (15)	0.10 (14)
ω_{12} (-)	–	–	0.16 (30)	–
ω_{21} (-)	–	–	0.79 (14)	–
σ_{add} (unit of biomarker)	biomarker	9.3 (15)	0.82 (20)	0.19(20)
σ_{prop} (%)	%	0.099 (3)	0.15 (4)	0.12 (5)

CK, creatine phosphokinase; cTnI, cardiac troponin I; CK-MB, creatine phosphokinase muscle-brain; ω, interindividual standard deviation; σadd; additive error standard deviation; σprop,

proportional error standard deviation; B_{TOT}, total biomarker input released by lesion. AAR, area at risk; ACS, abnormally contracting segments.

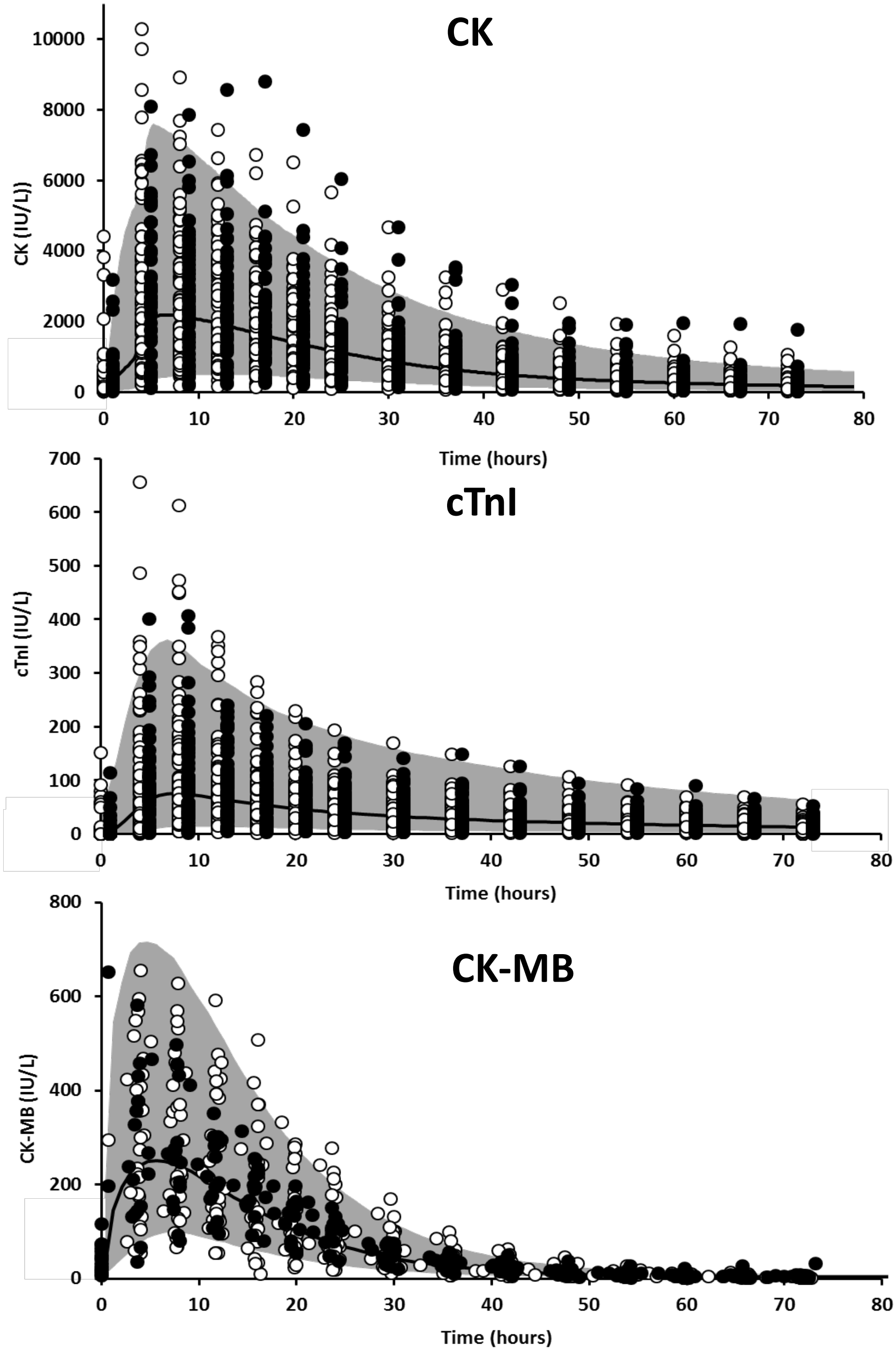

Supplementary figure 1. Prediction interval with 90% of kinetic profiles for creatine phosphokinase (CK), cardiac troponin I (cTnI) and creatine phosphokinase muscle-brain (CK-

MB) kinetic models *versus* time. White and black circles are observed biomarker levels of learning and validation subsets, respectively.

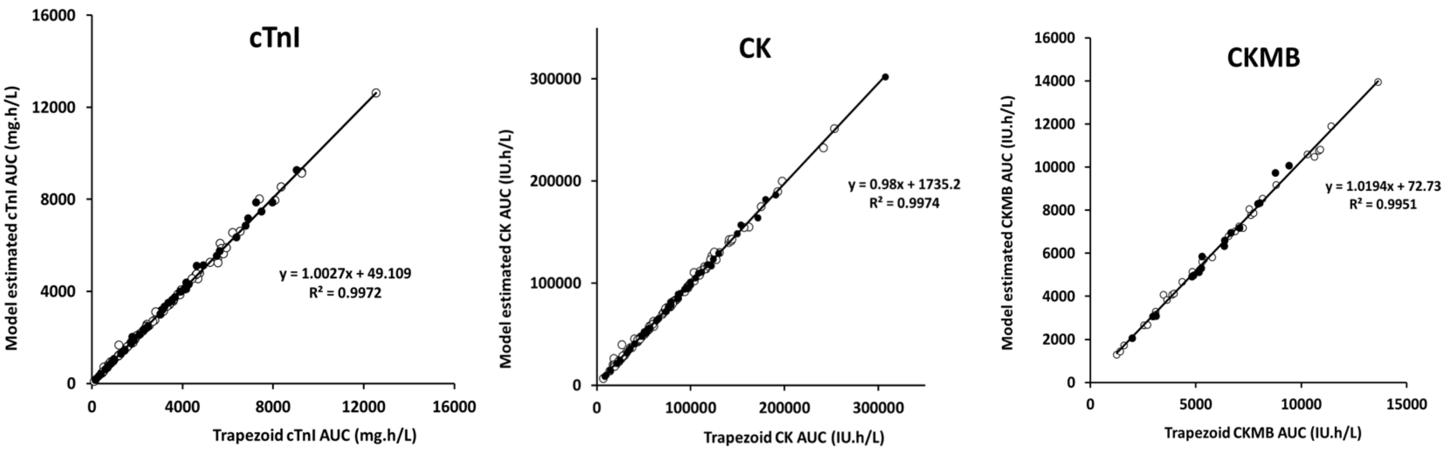

Supplementary figure 2. Linear correlations between model-predicted area under the biomarker curve (AUC) and trapezoidal rule AUC for creatine phosphokinase (CK, left), cardiac troponin I (cTnI, middle) and creatine phosphokinase muscle-brain (CK-MB). All correlation coefficients are above 99%. White and black circles are trapezoid/model-estimated AUC of learning and validation subsets, respectively.

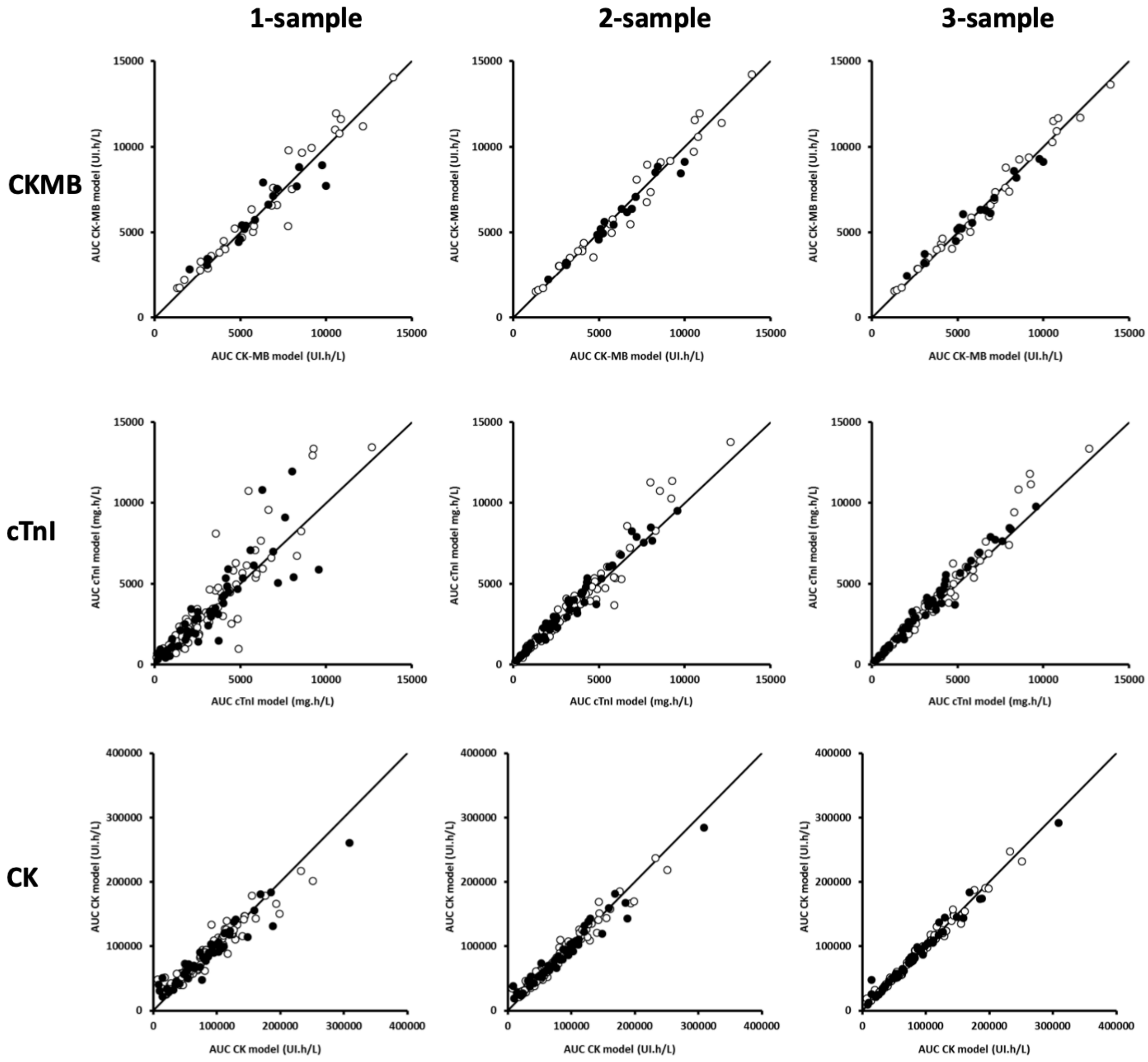

Supplementary figure 3. Observed versus model-predicted creatine kinase muscle-brain (CKMB, up), cardiac troponin I (cTnI, middle) and creatine kinase (CK, bottom) for limited sampling strategies. From left to right, all data points, then best 1-sample, 2-sample and 3-sample limited sampling strategies. Open and dark circles are observed/predicted biomarker level couples for learning an validation sets, respectively, the line is the first bisector line.

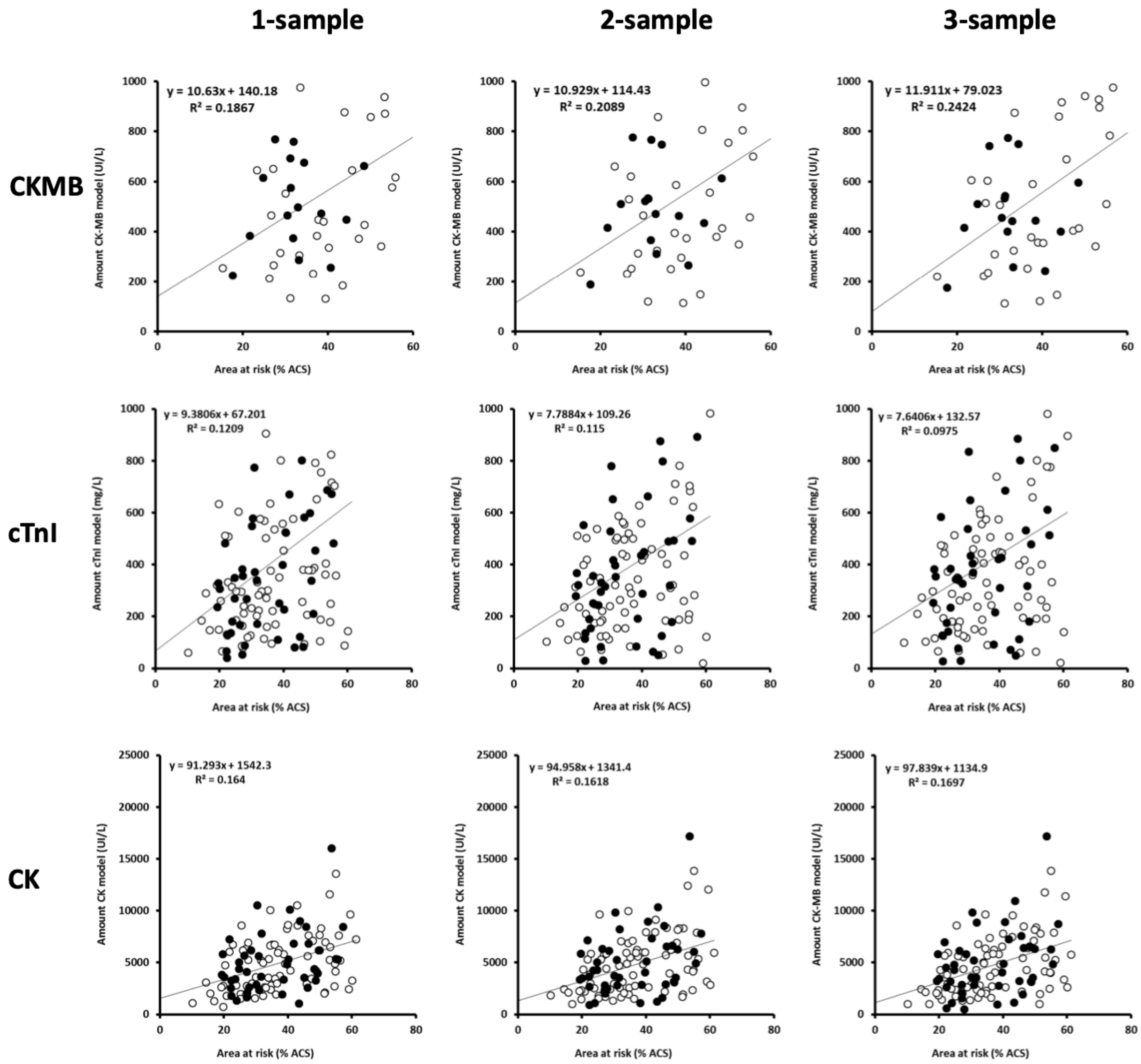

Supplementary figure 4. Area at risk of ischemic myocardium versus model-predicted creatine kinase muscle-brain (CK-MB, up), cardiac troponin I (cTnI, middle) and creatine kinase (CK, bottom) for models

From left to right, best 1-sample, 2-sample and 3-sample limited sampling strategies.

ACS, abnormally contracting segments.

SUPPLEMENTARY METHODS

1. Biomarker kinetic analysis

1.1. Patients population and data collection

Patients population and data collection were described previously in details[1]. Data were obtained from prospective studies in ST-segment elevation myocardial infarction (STEMI) patients involved in five clinical trials that were approved by local ethics committees and in which all patients had given written informed consent. The study was approved by the institutional review board of the Pole Coeur Thorax Vaisseaux from the Tours University Hospital (Tours, France), and was registered as a clinical audit. Ethical review was, therefore, not required. Patient consent was not sought. Patient data were used only to facilitate the cross-referencing of data sources, and records were otherwise anonymous. The present study was conducted retrospectively, patients were not involved in its conduct, and there was no impact on their care. Studies were:

- "PC" studies (n=33 and 38)[2,3]
- "PC CsA" (n=58)[4]
- "PCNR" (n=62)[5,6]
- "RIPOST-MI" (n=55)[7]

These were prospective, randomized, multicenter, open-label controlled studies that included STEMI patients who were referred for reperfusion by primary percutaneous intervention within 12 hours of the onset of chest pain and had a TIMI 0/1 coronary blood flow at first culprit coronary artery injection. In "PC", "PC CsA", and "PCNR" studies, patients were allocated to control or conditioning treatment arms, i.e. ischemic post-conditioning for "PC" and "PCNR", and pharmacological post-conditioning with Cyclosporine A for "PC CsA". Primary endpoints were infarct size estimated by area under the concentration versus time curve (AUC) of creatine kinase (CK) and Troponin I (cTnI), which was estimated using serial serum CK and cTnI measurements. In "RIPOST-MI" study, patients were randomly allocated to the control group, remote ischemic per-conditioning (RIPer) group, or RIPer + local ischemic post-conditioning (IPost) group. Primary endpoint was infarct size estimated by AUC of CK-MB estimated using serial serum measurements. In all studies, the area at risk of ischemic myocardium (AAR) was

estimated using a biplane left ventricular angiography in order to measure the circumferential extent of abnormally contracting segments (ACS)[8–11]. Blood samples were collected prior to primary percutaneous coronary intervention, every 4 h in the first 24 h following intervention, and every 6 h over the following 48 h. Therefore, 15 samples (T0, T4, T8…, T72) were available for each patient and biomarker. These samples had been used to compute AUC toward 72 hours for every patient individually, for CK (AUCCK), cTnI (AUCcTnI) and CK-MB (AUCCK-MB).

Until march 5th 2007 (54 patients), non-high-sensitive cTnI concentrations were measured using Accu kit on Access 2 system (Beckman Coulter, Villepinte, France) and CK concentrations were measured using CK reactive kit on Synchron LX system (Beckman Coulter). Starting from march 6th 2007 (78 patients), non-high-sensitive cTnI concentrations were measured using STAT ARCHITECT kit on Architect I2000 system (Abbott, Rungis, France) and CK concentrations were measured using Abbott 7D63 CK kit on architect C8000 and C16000 systems (Abbott). Since CK and cTnI concentrations were measured using two distinct assays, CK and cTnI concentrations, as well as kinetic parameters were compared between assays. No significant differences in concentrations or kinetics parameters were found between Beckman Coulter and Abbott kits. This was awaited since their performances were comparable. Notably, regarding cTnI, these two kits targeted epitopes 24-40 and 41-49 in common, epitopes 87-91 being targeted only by Abbott assay[12]. The kits had close characteristics: similar limits of detection (0.01 ng/mL), 99th percentile concentration (0.040 ng/mL and 0.028 ng/mL for Beckman Coulter and Abbott, respectively), coefficient of variation of 99th percentile (14%). Since at least 20 cTnI measurement assays are available with various performances, the validation of our kinetic model for cTnI assay kits (notably high-sensitive cTnI assays) is needed. However, we are convinced of the ability of our model to describe cTnI kinetic data obtained from various assays since Bayesian estimation of kinetic parameters is based on mixed-model effect modelling, which allows to take into account variability due to non-controlled or unknown factors, including differences in measurement techniques. This was confirmed with our external validation cohort. The 103 STEMI patients of this cohort had anterior STEMI with an initial TIMI 0/1 blood flow and were treated within 12 hours of the onset of chest pain by primary angioplasty[13]. All had non-hs and hs cTnI serial measurements within the first 72 hours following admission and primary angioplasty. Non-hs cTnI were measured using the Advia Centaur Ultra cTnI assay on an Advia Centaur

Immunoassay analyser from Siemens, with a 10% coefficient of variation limit of 30 ng/mL and a 99th percentile of 40 ng/mL (confidence interval, 20-60). Hs cTnI were measured using the Architect STAT High Sensitive Troponin-I Reagent on an Architect i1000SR from Abbott Diagnostics. The 10% coefficient of variation limit was 3.9 ng/mL and the 99th percentile values were 14 ng/mL for men and 11 ng/mL for women. Both assays were double chemiluminescent immunoassay where the capture antibody targeted epitope 41-49 and 24-40 of cTnI and the chimeric detection antibody targeted epitope 27-40 and 41-49 (for the non-hs and the hs cTnI assay respectively.

1.2. Kinetic models

The objective of kinetic analysis was to estimate the input of biomarker released by the lesion. Kinetics of total creatine phosphokinase (CK), muscle-brain CK (CK-MB) and Troponin Ic (cTnI) was described using models derived from commonly used pharmacokinetic compartmental models[14,15]. The kinetics of these biomarkers was developed and its parameters estimated separately, i.e. separate models were used for each biomarker. These models describe absorption, distribution, metabolism and excretion ("ADME") steps. Using these models, drugs are described as being distributed in a central compartment, corresponding to bloodstream and loci rapidly equilibrating with it, and one or more peripheral compartments, corresponding to other loci, which are not immediately in equilibrium with central compartment.

Biomarker release. Similarly to drug absorption (i.e. the passage from absorption site to bloodstream), the release of biomarkers by injured tissue (i.e. the passage of biomarker from tissue to bloodstream) is delayed in time. This non-instantaneous release may be described using models that are sometimes used to describe drug absorption. Among these models, transit absorption models[16] have been used to describe a delay in absorption occurring as drugs travel through a certain number (non-integer) of "transit compartments", as it is the case for CsA[17], mycophenolate[18] or rifampicin[19]. This transit model was already used to describe the kinetics of S100 calcium-binding protein, a biomarker used in traumatic brain injury[20]. For each patient, the origin of time is set to the first blood sample (t_0) and biomarker release function f(t) is written as follows:

$$f(t) = \frac{(k_{tr}.t)^n.e^{-k_{tr}.t}}{\Gamma(n)}$$

where n is the number (not necessarily integer) of transit compartments, k_{tr} is the transit rate constant, t is the time from the first blood sample and Γ(n) is gamma function. The release model R(t) may be:

- either a single transit model : R(t) = B . f(t)
- or a combination of two transit models (similarly to the absorption of mycophenolate) as follows:

$$R(t) = [F.f_1(t) + (1 - F).F_2(t)]$$

where B is the input of biomarker released for $t > t_0$, F is the proportion of biomarker amount released as described by $f_1(t)$, the first release model, and $f_2(t)$, the second release model. Both $f_1(t)$ and $f_2(t)$ are release functions with n_1 and n_2 transit compartments, respectively, and k_{tr1} and k_{tr2} are their respective transit rate constants. The parameters n and k_{tr} (for the single release function), or n_1, n_2, k_{tr1}, k_{tr2} and F (for the combination of two release functions) are structural parameters that had to be estimated.

The main parameter, which is the total input of biomarker released, was the sum of the biomarker already released before t_0 (B_0), and of the one released after t_0 (B). Under the assumption of homogenous distribution of biomarkers in bloodstream, this parameter is proportional to the total amount of biomarker (B_{TOT}), i.e. $B_{TOT} = B_0 + B$.

The mono-compartment model is written as follows:

$$\frac{dB}{dt} = R(t) + k_{prod} - k_e.B$$

where B is the biomarker input after t_0, f(t) is the release function, k_e is the elimination rate constant and k_{prod} zero-order (constant infusion) is the baseline biomarker production (i.e. independent from disease). Initial value of B(0) could not be given the value of zero because biomarker release started before the first blood sample and was $B(0) = B_0$. In this model, k_{prod}, k_e and B_0 are structural parameters that had to be estimated.

The two-compartment model is written as follows:

$$\frac{dB_1}{dt} = R(t) + k_{prod} - (k_e + k_{12}).B_1 + k_{21}.B_2$$

$$\frac{dB_2}{dt} = k_{12}.B_1 + k_{21}.B_2$$

where B_1 and B_2 are biomarker levels in central and peripheral compartments respectively, f(t) is the release function, k_{12} and k_{21} are the central to peripheral, and peripheral to central distribution rate constants. Initial values $B_1(0)$ and $B_2(0)$ (non-zero) had to be estimated as typical parameters. The amount of biomarker released before t_0 is $B_0 = B_1(0) + B_2(0)$. Similarly to the mono-compartment model, k_{12}, k_{21}, $B_1(0)$ and $B_2(0)$ are structural parameters that had to be estimated.

1.3. Model development

The development of kinetic models was made on learning subset.

Population approach. Population modelling has been used to describe pharmacokinetic data since the early 70's. If individual modelling is used to estimate the parameters of interest (*e.g.* the drug clearance in pharmacokinetics) at the "individual level", *i.e.* for each individual taken separately from the others, the basic principle of population modelling is to estimate parameters at the "population level"[15]. The main goals of population modelling are to determine the distribution of the values of the parameters of interest in a population and to quantify the influence of the individual sources of variability in this population. Using a population approach, data from all individuals in a given population are computed simultaneously to estimate the interindividual distribution of parameters of interest. This interindividual distribution allows the quantification of (i) the "mean" (referred as "typical") value of each parameter, (ii) the interindividual variability (referred as "interindividual variance") and (iii) the influence of individual factors on interindividual variability (referred as "covariates").

Software. Biomarker kinetic data were analysed by a population approach using the nonlinear mixed-effects program MONOLIX 4.3.2 software (Lixoft, Saclay, France), which combines the stochastic expectation-maximization (SAEM) algorithm and a Markov Chain Monte-Carlo procedure for likelihood maximization. To ensure the best possible convergence, a large number of iterations (1000 for K1 and 250 for K2) was used. K1 and K2 refer to the SAEM procedure of

Monolix, called “iterative kernels”. During K1, the sequence of step sizes is constant, which allows the exploration of the parameter space. During K2, the step sizes decrease to ensure convergence. Five Markov chains were used, and simulated annealing was used to improve the convergence of the SAEM algorithm towards the global maximum of the likelihood. Each run was performed three times to ensure that estimated parameters and likelihood remained stable. The random seed was changed between each of the three runs.

Structural model design. Biomarker concentrations were described with compartmental models with transit gamma release. One or two compartment models with first-order distribution constants were first tested. Structural models were compared using Akaike’s information criterion (AIC), defined as: AIC = OFV + 2.p, where OFV is the value of the objective function and p is the number of model parameters to estimate. The use of AIC is based on the parsimony principle aiming at a satisfactory fitting of the data with a small number of parameters. The OFV was –2.ln-likelihood (–2LL). The model with the lowest AIC was selected.

Interindividual model. The interindividual variability of pharmacokinetic parameters was described using an exponential model: $\theta_i = \theta_{TV} . \exp(\eta_i)$, where θ_i is the estimated individual parameter, θ_{TV} is the typical value of the parameter and η_i is the random effect for the ith patient. The values of η_i were assumed to be normally distributed with mean 0 and variance ω^2.

Error model. Additive, proportional and mixed additive-proportional models were tested. For example, the combined additive-proportional model was implemented as follows: $Y_{O,ij} = Y_{P,ij}.(1+ \varepsilon_{prop,i}j) + \varepsilon_{add,ij}$ where $Y_{O,ij}$ and $Y_{P,ij}$ are observed and predicted j^{th} marker measurements for the ith patient, respectively, and $\varepsilon_{prop,ij}$ and $\varepsilon_{add,ij}$ are proportional and additive errors, which are assumed to follow a Gaussian distribution with mean 0 and variances $\sigma_{prop}{}^2$ and $\sigma_{add}{}^2$, respectively.

Covariates. The influence of three individual potential factors of variability on the distribution of B_{TOT} was tested:

- continuous covariate: AAR (% ACS)
- discrete covariates: conditioning therapies.

- PC, PC_CsA and PCNR trials: study arm was coded as 0 (reference, control arm) or 1 (conditioned arm, i.e. ischemic postconditioning for PC and PCNR and pharmacological postconditioning with CsA for PC_CsA)
- RIPOST-MI trial: study arm was coded as 0 (reference, control arm), 1 (remote ischemic preconditioning, RIPer) or 2 (RIPer + local ischemic postconditioning, IPost).

The influence of a discrete covariate (ARM) on θ_{TV} was implemented as follows:
- For the reference category: ARM=0, $\ln(\theta_{TV}) = \ln(\theta_{ARM}=0)$
- For another category: $\ln(\theta_{TV}) = \ln(\theta_{ARM}=0) + \beta_{ARM}=x$, where $\theta_{ARM}=0$ is the value of θ for the control arm, and $\beta_{ARM}=x$ is the xth value of θ_{TV} for the other category.

Area at risk was tested as follows: $\theta_{TV} = \theta_0 . \exp(\beta_{AAR}.AAR)$, where β_{AAR} quantifies the influence of AAR on θ.

Model comparison and covariate selection. Interindividual, residual and covariate models are chosen by comparing 2 nested models, one with parameters, the difference of OFV is calculated. The difference in OFV is tested using a likelihood ratio test (RLT), i.e. the difference of OFV is compared to a chi-square law. However, LRT tests can be used only if the 2 models are nested, i.e. one model is a particular case of the other one. The influence of a covariate is tested by comparing OFVs of a model including the covariate (M1) and of a model without the covariate (M0). The value of $OFV_{M1} – OFV_{M0}$) is assumed to follow a χ^2 distribution and is therefore compared to a chi-square law with one degree of freedom for a given alpha risk.
The influence of patient characteristics (covariates) was assessed in two steps:
- *Univariate step*. The influence of each factor on pharmacokinetic parameters associated with interindividual variability was tested. Covariates were separately included into the base model. Covariates showing a significant influence ($\alpha < 0.1$) were included in the model (full model).
- *Multivariate step*. A backward stepwise elimination was performed: the covariates of the full model were removed one by one. Covariates whose removal resulted in a statistically significant increase in the OFV ($\alpha < 0.01$) were retained in the model. In RIPOST-MI trial, three arms were available and three values were therefore available for each covariate. If two categories were not significantly different, they were merged.

1.4. Model estimation of area under the biomarker concentration versus time curve (AUC)

To confirm that our pharmacokinetic model provides AUC estimations similar to those calculated by trapezoidal rule, individual AUCs for all biomarkers were (i) calculated using trapezoidal rule and (ii) computed using individual model parameter estimates. Trapezoidal rule AUC and model-computed AUC were compared using the coefficient of determination (R^2).

2. Performances of biomarker kinetic models

Model building was made on learning subsets. Biomarker kinetic models allowed an estimation of total inputs of CK (CK_{TOT}), cTnI ($cTnI_{TOT}$) and CK-MB ($CK\text{-}MB_{TOT}$) levels. The kinetics of these models were described and quantified separately from each other (supplementary table 1). Elimination half-lives of CK, cTnI and CK-MB were computed using kinetic parameters estimates and were 11.2 h, 37.8 h and 7.2 h, respectively.

2.1. Base models for CK, cTnI and CK-MB

The kinetic models that were tested included a release model with a single transit or a combination of two transit models, and a distribution/elimination model with one or two compartments. Therefore, four models were finally tested. Baseline biomarker production was removed from the model if it could not be estimated.

- CK data were best described using a two-transit mono-compartment model;
- cTnI data were best described using a two-transit two-compartment model, which is consistent with a previous study which reported a biphasic elimination of cTnI and two peaks[13];
- CK-MB data were best described using a single transit mono-compartment model.

The best error model was mixed additive-proportional for all biomarkers. Levels of CK, cTnI and CK-MB were adequately described by the kinetic models (supplementary figure 1). Interindividual variances of some release model parameters could not be estimated and were

therefore fixed to 0. All other parameters were estimated with good precision (with relative standard errors < 30%).

2.2. Covariate models

Regarding the association between body weight, age, AAR, study arm, and the total input of biomarker released by the lesion CK_{TOT}, $cTnI_{TOT}$ and $CK\text{-}MB_{TOT}$, the univariate step showed that ($\alpha < 0.1$):

(i) CK_{TOT} increased with AAR (LRT=10.2, p=0.0014) and was lower in the conditioning treatment arms (LRT=4.5, p=0.034)

(ii) $cTnI_{TOT}$ increased with AAR (LRT=12.1, p=0.0061) and was lower in conditioning treatment arms (LRT=3.4, p=0.064).

(iii) $CK\text{-}MB_{TOT}$ increased with AAR (LRT=8.7, p=0.0032) and was lower in RIPer and/or IPOST treatment arms (LRT=6.7, p=0.0098)

The multivariate step showed that ($\alpha < 0.01$):

(i) CK_{TOT} increased with AAR (LRT=11.06, p=0.0009), and was lower in the conditioning treatment arms (LRT=8.4, p=0.0037);

(ii) $cTnI_{TOT}$ increased with AAR (LRT=9.0, p=0.0027) and was lower in the conditioning treatment arms (LRT=5.8, p=0.016);

(iii) $CK\text{-}MB_{TOT}$ increased with AAR (LRT=16.9, $p=3.8.10^{-5}$) and was lower in RIPer and/or IPOST treatment arms (LRT=12.0, p=0.00054). Because the RIPer arm was not significantly different from the RIPer+IPOST arm (p=0.89), these arms were merged: the reference category was control and RIPer arms and the other category was RIPer / RIPer+IPOST.

2.3. Internal validation

Internal validation was made on validation subsets. Concentration-time profiles and AUC values between learning and validation subset results (supplementary figures 1 and 2). This shows that both model structures and estimated parameter distributions allow satisfactory predictions of biomarker kinetic profiles for patients independent from learning data set.

2.4. Bayesian estimation, limited sampling strategies (LSS), and LSS Bayesian models

Bayesian estimation consists in combining:

- prior information about the kinetics of a given compound (mean, interindividual variances and covariate effects), which include prior estimates of kinetic parameters;

- concentration measurements of a given patient for whom individual kinetics have to be estimated. This patient is assumed to belong to the population of interest; in other words, the patient should be similar to the prior population (same disease, drug, etc.). The development of LSS models was made on learning subset. The following strategy was applied to CK, cTnI and CK-MB biomarkers (supplementary figure 1), in which LSS models with 1, 2 and 3 samples were assessed. Overall, 180 sampling strategies were tested for each biomarker. All of the 15 one-sampling strategies were tested (from T0 to T72), i.e. 15 strategies for each biomarker. For 2- and 3-sampling strategies, because T0 and T>48 hours led to poor prediction performance, sampling time combinations between T4 and T48 were tested, e.g. T4-T8, T8-T16… and T4-T8-T16, T4-T8-T24…, which led to test 45 and 120 combinations with 2 and 3 samples for each biomarker.

2.5. LSS model development

The interindividual distribution of parameter determined using nonlinear mixed-effect modelling in learning subset was used to develop LSS models in learning subset. For each LSS model, parameter values for each patient, as well as AUC, were estimated by fixing interindividual distribution as priors. LSS models were assessed using AUC, by comparing estimates of AUC obtained from each LSS model to those of kinetic model. Comparison of AUC values was made using coefficient of determination (R2) and relative bias. The best models with 1, 2 and 3 samples should have the highest R2 values with short sampling schedules (ideally sampling times ≤ 24 hours) and relative bias < 10%.

2.6. LSS model validation

The validation step was made in the validation subset. The interindividual distribution of parameters determined using nonlinear mixed-effect modelling in learning subset was used to estimate parameter values and AUC for each patient:

- kinetic model using all samples (T0 to T72);

- best LSS models with 1, 2 and 3 samples chosen as described above.

Performance of LSS models were evaluated by comparing:

- LSS model-estimated AUC values vs. kinetic model-estimated AUC values;
- LSS model-estimated AUC values vs. trapezoid-calculated AUCs;

These comparisons were made using R2 and bias as previously described.

References

1. Ternant, D. *et al.* Revisiting myocardial necrosis biomarkers: assessment of the effect of conditioning therapies on infarct size by kinetic modelling. *Sci Rep* **7**, 10709 (2017). DOI: 10.1038/s41598-017-11352-4
2. Staat, P. *et al.* Postconditioning the human heart. *Circulation* **112**, 2143–2148 (2005). DOI: 10.1161/CIRCULATIONAHA.105.558122
3. Thibault, H. *et al.* Long-term benefit of postconditioning. *Circulation* **117**, 1037–1044 (2008). DOI: 10.1161/CIRCULATIONAHA.107.729780
4. Piot, C. *et al.* Effect of cyclosporine on reperfusion injury in acute myocardial infarction. *N. Engl. J. Med.* **359**, 473–481 (2008). DOI: 10.1056/NEJMoa071142
5. Thuny, F. *et al.* Post-conditioning reduces infarct size and edema in patients with ST-segment elevation myocardial infarction. *J. Am. Coll. Cardiol.* **59**, 2175–2181 (2012). DOI: 10.1016/j.jacc.2012.03.026
6. Mewton, N. *et al.* Postconditioning attenuates no-reflow in STEMI patients. *Basic Res. Cardiol.* **108**, 383 (2013). DOI: 10.1007/s00395-013-0383-8
7. Prunier, F. *et al.* The RIPOST-MI study, assessing remote ischemic perconditioning alone or in combination with local ischemic postconditioning in ST-segment elevation myocardial infarction. *Basic Res. Cardiol.* **109**, 400 (2014). DOI: 10.1007/s00395-013-0400-y
8. Feild, B. J., Russell, R. O., Dowling, J. T. & Rackley, C. E. Regional left ventricular performance in the year following myocardial infarction. *Circulation* **46**, 679–689 (1972). DOI: 10.1161/01.cir.46.4.679

9. Rogers, W. J. *et al.* Correlation of angiographic estimates of myocardial infarct size and accumulated release of creatine kinase MB isoenzyme in man. *Circulation* **56**, 199–205 (1977). DOI: 10.1161/01.cir.56.2.199
10. Rigaud, M. *et al.* Regional left ventricular function assessed by contrast angiography in acute myocardial infarction. *Circulation* **60**, 130–139 (1979). DOI: 10.1161/01.cir.60.1.130
11. Lapeyre, A. C., St Gibson, W., Bashore, T. M. & Gibbons, R. J. Quantitative regional wall motion analysis with early contrast ventriculography for the assessment of myocardium at risk in acute myocardial infarction. *Am. Heart J.* **145**, 1051–1057 (2003). DOI: 10.1016/S0002-8703(03)00112-1
12. Christenson, R. H. *et al.* Comparison of 13 Commercially Available Cardiac Troponin Assays in a Multicenter North American Study. *The Journal of Applied Laboratory Medicine: An AACC Publication* **1**, 544–561 (2017). DOI: 10.1373/jalm.2016.022640
13. Laugaudin, G. *et al.* Kinetics of high-sensitivity cardiac troponin T and I differ in patients with ST-segment elevation myocardial infarction treated by primary coronary intervention. *Eur Heart J Acute Cardiovasc Care* **5**, 354–363 (2016). DOI: 10.1177/2048872615585518
14. Mould, D. R. & Upton, R. N. Basic concepts in population modeling, simulation, and model-based drug development. *CPT Pharmacometrics Syst Pharmacol* **1**, e6 (2012). DOI: 10.1038/psp.2012.4
15. Mould, D. R. & Upton, R. N. Basic concepts in population modeling, simulation, and model-based drug development-part 2: introduction to pharmacokinetic modeling methods. *CPT Pharmacometrics Syst Pharmacol* **2**, e38 (2013). DOI: 10.1038/psp.2013.14

16. Savic, R. M., Jonker, D. M., Kerbusch, T. & Karlsson, M. O. Implementation of a transit compartment model for describing drug absorption in pharmacokinetic studies. *J Pharmacokinet Pharmacodyn* **34**, 711–726 (2007). DOI: 10.1007/s10928-007-9066-0
17. Leger, F. *et al.* Maximum a posteriori Bayesian estimation of oral cyclosporin pharmacokinetics in patients with stable renal transplants. *Clin Pharmacokinet* **41**, 71–80 (2002). DOI: 10.2165/00003088-200241010-00006
18. Prémaud, A. *et al.* A double absorption-phase model adequately describes mycophenolic acid plasma profiles in de novo renal transplant recipients given oral mycophenolate mofetil. *Clin Pharmacokinet* **44**, 837–847 (2005). DOI: 10.2165/00003088-200544080-00005
19. Wilkins, J. J. *et al.* Population pharmacokinetics of rifampin in pulmonary tuberculosis patients, including a semimechanistic model to describe variable absorption. *Antimicrob. Agents Chemother.* **52**, 2138–2148 (2008). DOI: 10.1128/AAC.00461-07
20. Ercole, A., Thelin, E. P., Holst, A., Bellander, B. M. & Nelson, D. W. Kinetic modelling of serum S100b after traumatic brain injury. *BMC Neurol* **16**, 93 (2016). DOI: 10.1186/s12883-016-0614-3
